# Overexpression of c-Met in bone marrow mesenchymal stem cells improves their effectiveness in homing and repair of acute liver failure

**DOI:** 10.1186/s13287-017-0614-2

**Published:** 2017-07-05

**Authors:** Kun Wang, Yuwen Li, Tiantian Zhu, Yongting Zhang, Wenting Li, Wenyu Lin, Jun Li, Chuanlong Zhu

**Affiliations:** 1 Department of Infectious Disease, the First Affiliated Hospital with Nanjing Medical University, Nanjing, China; 2 Department of Infectious Disease, Anhui Provincial Hospital, Anhui Medical University, Hefei, China; 3 Department of Pediatrics, the First Affiliated Hospital with Nanjing Medical University, Nanjing, China; 4 Liver Center and Gastrointestinal Division, Department of Medicine, Massachusetts General Hospital, Harvard Medical School, Boston, Massachusetts USA

**Keywords:** c-Met, Bone marrow mesenchymal stem cells, Liver injury, Acute liver failure, Lentiviral vector

## Abstract

**Background:**

Transplantation of bone marrow-derived mesenchymal stem cells (BMSCs) has emerged as a novel therapy for acute liver failure (ALF). However, the homing efficiency of BMSCs to the injured liver sites appears to be poor. In this study, we aimed to determine if overexpression of c-Met in BMSCs could promote the homing ability of BMSCs to rat livers affected by ALF.

**Methods:**

Overexpression of c-Met in BMSCs (c-Met-BMSCs) was attained by transfection of naive BMSCs with the lenti-c-Met-GFP. The impact of transplanted c-Met-BMSCs on both homing and repair of ALF was evaluated and compared with lenti-GFP empty vector transfected BMSCs (control BMSCs).

**Results:**

After cells were transfected with the lenti-c-Met-GFP vector, the BMSCs displayed very high expression of c-Met protein as demonstrated by Western blot. In addition, in vitro transwell migration assays showed that the migration ability of c-Met-BMSCs was significantly increased in comparison with that of control BMSCs (*P* < 0.05), and was dependent on hepatocyte growth factor (HGF). Furthermore, rats with ALF that received transplanted c-Met-BMSCs showed significantly improved homing ability to the injured liver; this was accompanied by elevated survival rates and liver function in the ALF rats. Parallel pathological examination further confirmed that transplantation of c-Met-BMSCs ameliorated liver injury with reduced hepatic activity index (HAI) scores, and that the effects of c-Met-BMSCs were more profound than those of control BMSCs.

**Conclusions:**

Overexpression of c-Met promotes the homing of BMSCs to injured hepatic sites in a rat model of ALF, thereby improving the efficacy of BMSC therapy for ALF repair.

## Background

Acute liver failure (ALF) is the rapid loss of liver function due to severe damage to the liver, with common causative factors including viruses (particularly hepatitis B and C), toxins, prescribed drugs, and alcohol. ALF can lead to jaundice, coagulopathy, multiple organ failure, hepatic encephalopathy, and even death [1]. Currently, liver transplantation (LT) is considered the most effective therapy for this disease. However, its application for ALF is limited by a shortage of available donor organs and the procedure is invasive [2]. Bone marrow-derived mesenchymal stem cells (BMSCs) are multipotent stem cells that exhibit differentiation activity and significant potential for self-renewal. Furthermore, these cells can differentiate into a variety of cell types, including osteoblasts, chondrocytes, adipocytes, and hepatocytes. It has also been reported that transfection of exogenous genes and consequent protein expression appear to be readily manipulated in BMSCs [3]. Considering the aforementioned advantages of BMSCs, they have been employed for the repair of damaged tissues or organs including the liver. In fact, BMSCs have been shown to be effective in the treatment of hepatic cirrhosis and liver failure. In addition, studies with BMSCs have demonstrated that they can repair ALF by regulating inflammatory responses and secreting trophic factors such as hepatocyte growth factor (HGF) and basic fibroblast growth factor (bFGF). However the ability of BMSCs to home to the injured liver has been reported as being poor. Consequently, this has posed challenges for development of their application [4]. Given the significant morbidity and mortality associated with ALF, there is an urgent need to enhance the homing capabilities of BMSCs in order to improve the efficacy of these potent cell types.

c-Met is encoded by the *MET* gene and belongs to the tyrosine protein kinase family. The c-Met protein is a member of the transmembrane tyrosine kinase receptor superfamily and has independent phosphorylation activity [5]. HGF is commonly known as the ligand of c-Met. The HGF/c-Met signaling pathway is considered to play an important role in the homing ability of BMSCs to the liver, which permits stem cell-mediated repair of the liver. BMSCs have been shown to influence both the differentiation of BMSCs into hepatocytes, and liver regeneration [6].

In this study, we aimed to establish c-Met-BMSCs by overexpression of c-Met, and to determine if c-Met-BMSCs could promote homing of BMSCs to rat livers, thereby improving their capability for repairing ALF.

## Methods

### Animals

A total of seventy-two male Sprague-Dawley rats were purchased from the Animal Laboratory Center of Nanjing Medical University (Nanjing, China). Rats were aged 4 weeks, weighed 80–100 g, and were used to isolate BMSCs so that an ALF rat model could be generated. All rats were maintained according to the experimental animal care and research protocol, which was approved by the First Affiliated Hospital of Nanjing Medical University (Nanjing, China). All experiments on rats were carried out in compliance with the guidelines of the Chinese Ethical Council.

### Isolation, culture, and validation of rat BMSCs

Fresh rat BMSCs were isolated from male rats. Briefly, male rats were initially anesthetized by administration of 10% chloral hydrate at a dosage of 0.3 mL/100 g. The ends of the femur, tibia, and soft connective tissues were carefully removed to expose the intact bone marrow cavity, which was washed twice with normal saline solution, and collected into a centrifuge tube. Bone marrow material was centrifuged for 10 min at 180 × g. The pellets were then dissolved in complete cell culture medium containing a low glucose solution of 89% Dulbecco’s modified Eagle’s medium (DMEM; HyClone, Logan, USA), 10% fetal bovine serum (FBS; Corning, New York, USA), and 1% penicillin/streptomycin (Biyuntian, China). The mixture was suspended in lymphocyte separation medium (Gibco, New York, USA) and centrifuged for 20 min at 710 × g. The third layer was extracted, placed into a 10-cm^2^ culture dish with complete culture medium, and cultured in an incubator at 37 °C at an atmosphere of 5% CO_2_ with saturated humidity. BMSCs from the third passage were selected and then a total of 10^5^/100 μL cells were labeled with different antibodies, including FITC-labeled anti-rat CD29, APC-labeled anti-rat CD45, PE-labeled anti-rat CD90, and anti-rat CD34 (Becton, Dickinson company, USA). The solution was subsequently stored on ice for 30 min in the dark. After washing with phosphate-buffered saline (PBS), the cells were subjected to analysis and validation of specific markers by flow cytometry.

### Animal model of acute liver failure

Male rats were selected to generate the animal model of ALF as previously described [7]. In the protocol, rats received simultaneous intraperitoneal (i.p.) injections of d-galactosamine (d-GalN) at 950 mg/kg body weight (Sigma, St. Louis, MO, USA) and lipopolysaccharide (LPS) at 10 μg/kg body weight (Sigma, St. Louis, MO, USA).

### Detection of HGF levels by enzyme-linked immunosorbent assay (ELISA)

The heart, liver, spleen, lung, and kidneys of rats were collected at different time points (0, 24, and 48 h) post-d-GalN/LPS injection; 50 mg of each tissue was homogenized with 1.5 mL of lysis buffer (IS007-2, Cloud-clone Corp., China) and the lysate was collected to measure the HGF levels of all tissue samples using the HGF ELISA kit (Cloud-clone Corp., China), following the manufacturer’s instructions.

### Construction and transfection of the lenti-c-Met-GFP and control lenti-GFP empty vectors

For application of c-Met by polymerase chain reaction (PCR), a pair of primers were designed and synthesized as follows: Forward primer: 5’-GAGGATCCCCGGGTACCGGTCGCCACCATGAAGGCTCCCACCGCGCTGGCACCTGG-3’; Reverse primer: 5’-TCCTTGTAGTCCATACCTGTGTTCGCTTCGCCGTCAATGTTGTCTTG-3’. The resulting c-Met cDNA was inserted into the lentiviral vector GV358 (sequence elements: Ubi-MCS-3FLAG-SV40-EGFP-IRES-puromycin; Shanghai Genechem Company, China) to create the lentiviral vector, GV358-c-Met. We cotransfected the recombinant and two lentiviral helper plasmids (Helper1.0 and Helper2.0; Shanghai Genechem Company, China) into 293 T cells to generate the target lentivirus with an infectious viral titer of 2 × 10^8^ TU/mL, which was measured using a fluorescence assay method. In parallel, the control lentivirus was produced by cotransfecting the lenti-GFP empty vector GV358 with GFP but without c-Met, and two lentiviral helper plasmids (Helper1.0 and Helper2.0) into 293 T cells, and an infectious viral titer of 1 × 10^8^ TU/mL was obtained.

### Preparation of c-Met-BMSCs and control BMSCs

To establish c-Met-BMSCs or control BMSCs, the lenti-c-Met-GFP or lenti-GFP empty vector was used to transfect naive BMSCs cells at passage 5 (MOI = 100), respectively. Three days after infection, c-Met-BMSCs or control BMSCs were selected and cultured in cell culture medium containing 9 μg/mL puromycin for 2 weeks to generate stable cell lines (Patent﻿ no.20161066﻿2140.4). c-Met-BMSCs or control BMSCs were visualized under fluorescence microscopy to confirm a 100% fluorescence positive rate, and cells were screened and selected by puromycin.

### Western blot analysis of c-Met protein

Western blot (WB) analysis was performed to examine levels of c-Met protein, in which the same number of c-Met-BMSCs and control BMSCs (transfected with lenti-GFP empty vector) were used to extract total protein samples. Proteins were then separated by sodium dodecyl sulfate-polyacrylamide gel electrophoresis (SDS-PAGE) and transferred onto nitrocellulose membranes as previously reported [6]. The c-Met protein was detected with the rabbit anti-c-Met (SP260) primary antibody (Santa Cruz Biotechnology, Santa Cruz, USA) with mouse anti-actin antibody as control antibody (Santa Cruz Biotechnology, Santa Cruz, USA) for detection of the internal control protein, beta-actin. The secondary antibodies used in the WB analysis were a horseradish peroxidase (HRP)-conjugated donkey anti-rabbit IgG and a HRP-conjugated ECL sheep anti-mouse IgG (GE Healthcare Biosciences, UK). The resulting blots were detected by enhanced chemiluminescence (ECL) using an Amersham ECL Western blot detection kit (GE Healthcare Biosciences, Pittsburgh, USA) and visualized under an imaging system.

### Cell migration assay

The cell migration assay was performed in a transwell chamber (Corning, New York, USA) which contains a polyethylene terephthalate (PET) track-etched membrane with an 8.0-μm pore; 0.5 mL suspensions of c-Met-BMSCs or control BMSCs at a concentration of 1 × 10^5^ cells/mL were added to the top of the chamber layer. In the bottom chamber, different concentrations of murine HGF (Peprotech, USA) were used as a chemoattractant. The above cell transwell chambers were incubated in a humidified tissue culture incubator overnight at 37 °C and an atmosphere of 5% CO_2_. After 24 h, the cells were fixed and stained with 4% paraformaldehyde and 0.1% crystal violet, respectively. The migrated cells were observed, imaged, and counted within three fields under an optical microscope.

### Transplantation of c-Met-BMSCs and control BMSCs

A total of 36 rats were randomly divided into three experimental groups: c-Met-BMSC group, control BMSC group, and normal saline (NS) group, with twelve rats in each group. The rat model of ALF was induced and generated as previously described by co-injection of d-GalN/LPS. After 24 h, rats in the c-Met-BMSC and control BMSC groups were given a transfusion of 1.0 × 10^7^/kg cells, suspended in 1 mL normal saline by vena caudalis injection, respectively. In the NS group, animals were injected with 1 mL of normal saline. The survival of rats in the three groups were observed and recorded daily. The blood samples of all rats were collected at different time points (0, 24, 48, and 72 h) post-injection of d-GalN/LPS. The liver tissues of all the rats in the three groups were collected at 24, 48, or 72 h post-injection of d-GalN/LPS.

### Evaluation of liver necroinflammatory activity by serum ALT, AST, TBil, and hematoxylin-eosin staining

The amount of inflammation in the liver was quantified by assessing serum levels of alanine transaminase (ALT), aspartate aminotransferase (AST), total bilirubin (TBil), and hematoxylin-eosin (HE) staining. Serum levels of ALT, AST, and TBil were detected using a microplate test kit from Nanjing Jiancheng bioengineering institute (Nanjing, China). Rat liver tissues obtained from all three experimental groups were fixed in 4% paraformaldehyde, embedded in paraffin, and sliced to a thickness of 4 μm. All slices were subsequently stained by HE. The pathological images were captured and grades of liver inflammation were assessed by hepatic activity index (HAI) grading following guidelines as previously described [8].

### Analysis of BMSC homing ability to rat liver

To compare the homing efficiency of c-Met-BMSCs and control BMSCs to the injured rat liver, the dye DiR (AAT Bioquest, USA) was used to label both types of BMSCs, with or without c-Met overexpression; 1 × 10^6^ cells from each group were transplanted into the rat with ALF through the vena caudalis. After 24 h, migration of cells into the liver was examined using an in vivo imaging system (Animal Core Facility of Nanjing Medical University, Nanjing, China), and the homing efficiency was assessed by visualization of fluorescent intensity.

### Statistical analysis

The experiments in this study were performed with triplicate samples in each group. Statistical analysis was conducted using IBM Statistics SPSS, version 16.0 (SPSS, Chicago, IL, USA). Data are expressed as means ± standard deviation (SD). The log rank test was used to compare survival rates among the three groups, and statistical analysis was carried out using one-way analysis of variance. *P* values less than 0.05 were considered to be statistically significant.

## Results

### Preparation and characterization of BMSCs and c-Met-BMSCs

**Fig. 1:**
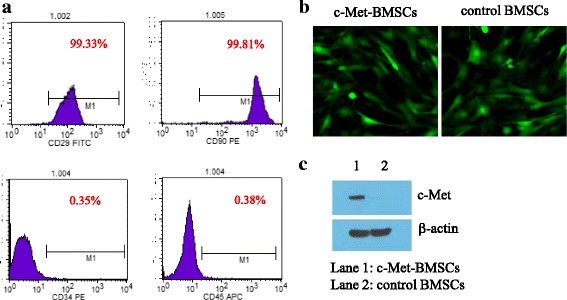
Overexpression of c-Met in BMSCs transfected with lenti-c-Met-GFP vector. The BMSCs were stably transfected with lenti-c-Met-GFP or lenti-GFP empty vector, and then analyzed for c-Met protein expression. **a** Determination of the naive bone marrow-derived mesenchymal stem cell (*BMSC*) phenotypes by flow cytometry. **b** Transfection efficiency was detected by fluorescence microscopy (×400) in c-Met-BMSCs and control BMSCs. **c** Western blot analysis of c-Met protein expression in c-Met-BMSCs and control BMSCs

Seven to ten days after isolated BMSCs were cultured the cell morphology was examined under an inverted microscope. Monolayers of BMSCs were formed with cells displaying a spindle shape and arranged in radial concentric circles or with broom-like growth (data not shown). Next, we characterized and validated the BMSCs at passage 3 by flow cytometry using specific cell surface markers for BMSCs. As shown in Fig. 1a, 0.35% of cells tested CD34-positive with 0.38% of cells being CD45-positive; 99.33% of the cells were CD29-positive and 99.81% were CD90-positive, indicating that the specific surface markers of the BMSCs had increased considerably in the prepared BMSCs. All results were consistent with the previous study by Li et al. [9]. Both the morphology shift and the fact that the surface markers of BMSCs were highly prevalent validated and confirmed the successful isolation of the BMSCs.

After naive BMSCs at passage 5 were transfected with the lenti-c-Met-GFP or lenti-GFP empty vector, and puromycin selection was completed, approximately 99% of these BMSCs were tested positive for green fluorescence as analyzed by fluorescence microscopy (Fig. 1b), indicating successful genetic modification of the BMSCs. We also observed that the expression of c-Met declined with an increase in the passage number of naive BMSCs (data not shown). Western blot analysis revealed that c-Met protein was markedly overexpressed in c-Met-BMSCs compared with control BMSCs (Fig. 1c), demonstrating the successful establishment of the c-Met-BMSC cell line with stable c-Met overexpression.

### Induction of HGF by the injured liver post-d-GalN/LPS injection in rats

**Fig. 2:**
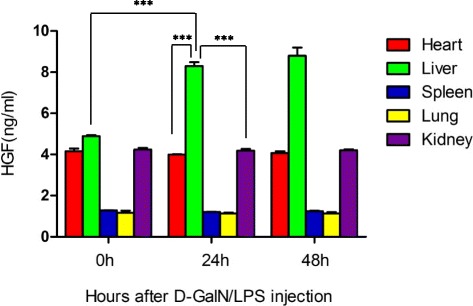
The concentrations of HGF in the heart, liver, spleen, lung, and kidneys at different time points post-coinjection of d-GalN/LPS. The heart, liver, spleen, lung, and kidney tissues were collected at 0, 24, and 48 h post-coinjection of d-GalN/LPS; 50 mg of each tissue was homogenized in 1.5 mL lysis buffer, and the lysate was collected for measurement of the concentrations of HGF by ELISA. Data are presented as mean ± SD. ***P < 0.001. *d* *-GalN* d-galactosamine, *HGF* hepatocyte growth factor, *LPS* lipopolysaccharide

As HGF is known to play an essential role in HGF/c-Met signaling-mediated repair of the injured liver, we examined the concentrations of HGF in the liver at 0, 24, and 48 h post-d-GalN/LPS injection. We also performed a parallel examination of HGF levels in other organs, including the heart, spleen, lung, and kidneys of the rat. The HGF concentrations at 0 h post-d-GalN/LPS injection were 4.89 ± 0.08 ng/mL, 4.16 ± 0.22 ng/mL, 1.29 ± 0.02 ng/mL, 1.17 ± 0.17 ng/mL, and 4.24 ± 0.145 ng/mL in the liver, heart, spleen, lung, and kidneys, respectively. We found that levels of HGF in the liver significantly increased after 24 h, with a peak level at 48 h post-d-GalN/LPS injection. However, there were no significant alterations in levels of HGF in the heart, spleen, lung, and kidneys of rats after d-GalN/LPS injection, which corroborates previous findings that HGF is induced by liver injury. As shown in Fig. 2, there was no statistical difference in HGF levels between these organs prior to d-GalN/LPS injection, whereas HGF levels in the liver were significantly higher compared with other organs at 24 h post-d-GalN/LPS injection (*P* < 0.001). These data indicate that there was induction of HGF through liver injury.

### Effects of c-Met overexpression on BMSC migration induced by HGF

**Fig. 3:**
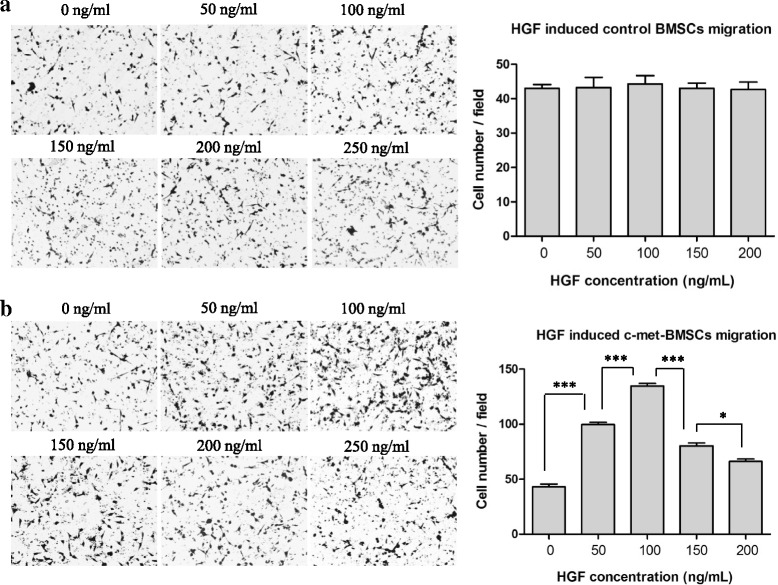
Transwell migration assays of c-Met-BMSCs versus BMSCs. Transwell migration assays were carried out to compare the migration ability of c-Met-BMSCs versus control BMSCs as described in the Methods section. The migrated cells were visualized and calculated under light microscopy. **a** Control bone marrow-derived mesenchymal stem cells (*BMSCs*) and **b** c-Met-BMSCs migration induced by different concentrations of murine hepatocyte growth factor (*HGF*). Data are presented as mean ± SD. *P < 0.05, ***P < 0.001

Next, we performed in vitro transwell migration assays to determine if the migration ability of c-Met-BMSCs could be improved in comparison with control BMSCs. The migrated BMSCs were visualized and counted under the microscope. The number of migrated cells in the c-Met-BMSC group were 43.0 ± 4.6, 100.7 ± 2.1, 127.3 ± 2.5, 80.3 ± 4.5, 66.0 ± 4.0, and 61.0 ± 3.6, respectively, while the number of migrated cells in the control BMSC group were 43.0 ± 2.0, 43.3 ± 5.0, 44.33 ± 1.2, 43.3 ± 2.1, 44.0 ± 3.0, and 43.3 ± 2.5, respectively. As shown in Fig. 3a, there was no significant difference in migrating cell numbers in the control BMSC group at different concentrations of HGF in the transwell chamber. However, at levels of HGF lower than 100 ng/mL the number of migrated cells in the c-Met-BMSC group increased in parallel with an increase in the concentrations of HGF (Fig. 3b). Interestingly, when the concentration of HGF was greater than 100 ng/mL the number of migrated cells actually decreased (Fig. 3b).

### Enhanced ability of homing in c-Met-BMSCs to the liver of rats with ALF

**Fig. 4:**
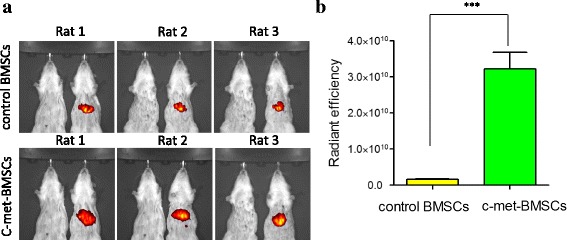
Analysis of cell migration in ALF rats transplanted with c-Met-BMSCs using an in vivo imaging system. DiR dye was used to label the c-Met-BMSCs and control BMSCs. The same amount of c-Met-BMSCs and control BMSCs were transplanted into ALF rats through the vena caudalis. After 24 h cells that had migrated to the injured liver were detected by an imaging system, and the fluorescent intensity was measured. **a** The fluorescent intensity of the liver tissues in rats transplanted with control bone marrow-derived mesenchymal stem cells (*BMSCs*) or c-Met-BMSCs. **b** The difference between the two groups was statistically significant. Data are presented as mean ± SD. ***P < 0.001

As described above, enhanced in vitro migration of c-Met-BMSCs may improve their homing ability to the injured liver. We therefore assessed effects of overexpressed c-Met protein on the homing capability of c-Met-BMSCs to the liver of rats with ALF. Control BMSCs and c-Met-BMSCs were labeled with the dye DiR and then transplanted into the liver of rats with ALF. Fluorescence was released from DiR-labeled cells in affected organs, and the fluorescent intensity was measured using an imaging system (Fig. 4a). As shown in Fig. 4b, the fluorescent intensity was (1.61 ± 0.25) × 10^9^ in the liver of ALF rats transplanted with control BMSCs, and (3.22 ± 0.80) × 10^10^ in the liver of ALF rats transplanted with c-Met-BMSCs, which was significantly higher than that in the control BMSC group (this represented a fold-change of 19.84 ± 1.71, *P* < 0.001). These data demonstrated that c-Met-BMSCs could migrate more efficiently into the liver of ALF rats compared with control BMSCs.

### Effects of c-Met-BMSC transplantation on survival rates and liver function in ALF rats

**Fig. 5:**
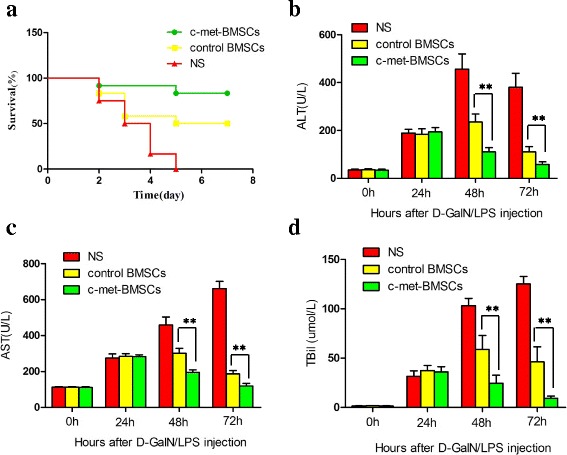
Effects of c-Met-BMSC transplantation on the survival rates and liver function in ALF rats. A total of 54 rats were randomly divided into three groups: c-Met-BMSC, control BMSC, and NS groups (*n* = 12 in each group). The rats were injected with d-GalN/LPS to induce ALF. After 24 h, the c-Met-BMSC and control BMSC groups were given a transfusion of 1.0 × 10^7^/kg cells suspended in 1 mL of normal saline (*NS*) respectively, while the NS group was given 1 mL of NS. The blood samples were collected at 0, 24, 48 and 72 h post-d-GaIN/LPS injection. **a** The survival rates in the c-Met-BMSC, control BMSC, and NS groups; levels of serum **b** alanine transferase (*ALT*), **c** aspartate aminotransferase (*AST*), and **d** total bilirubin (*TBil*) in treatment of ALF rats. Data were expressed as mean ± SD. *P < 0.05, **P < 0.01, and ***P < 0.001. *BMSC* bone marrow-derived mesenchymal stem cell, *d* *-GalN* d-galactosamine, *LPS* lipopolysaccharide

To evaluate the therapeutic effectiveness of c-Met-BMSCs for treatment of ALF in rats, we established a rat model of ALF. The rats with ALF were randomly divided into three groups: the c-Met-BMSC group, the control BMSC group, and the NS group. The survival rates were 83.3%, 50%, and 0% in the c-Met-BMSC, control BMSC, and NS groups, respectively (Fig. 5a). The ALF rats transplanted with c-Met-BMSCs had significantly higher survival rates than ALF rats treated with control BMSCs or NS (*P* < 0.05). In addition, we tested the serum levels of ALT, AST, and TBil at 0, 24, 48, and 72 h post-d-GalN/LPS injection. As shown in Fig. 5b-d, liver function started deteriorating 24 h after d-GalN/LPS injection. However, there was no significant difference in serum levels of ALT, AST, and TBil among the three groups. The serum levels of ALT, AST, and TBil were significantly lower in the c-Met-BMSCs group compared with NS group, at 48 h or 72 h after d-GalN/LPS injection (*P* < 0.001). Moreover, significant differences in serum levels of ALT, AST, and TBil were observed between c-Met-BMSCs and control BMSCs groups at both 48 and 72 h post-d-GalN/LPS injection (*P* < 0.01).

### Effects of c-Met-BMSC transplantation on liver function through evaluation of liver pathology in rats with ALF

**Fig. 6:**
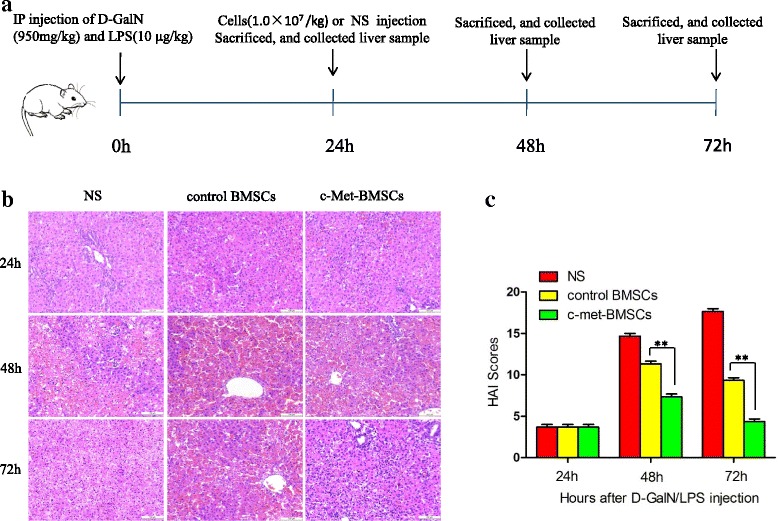
Effects of c-Met-BMSC transplantation on liver pathology of rats with ALF. Eighteen rats were randomly divided into three groups: c-Met-BMSC, control BMSC, and NS groups. **a** Schematic representation of the experimental procedures. **b** Liver tissues from the different groups were collected at 24, 48, and 72 h after d-GaIN/LPS injection and subsequently HE stained. **c** Histopathological grading of necrosis and inflammation of the liver sections. Data are presented as mean ± SD. **P < 0.01. *BMSC* bone marrow-derived mesenchymal stem cell, *d* *-GalN* d-galactosamine, *HAI* hepatic activity index, *IP* intraperitoneal, *LPS* lipopolysaccharide, *NS* normal saline

We further examined whether c-Met-BMSC transplantation could improve repair of ALF as assessed by liver histology. Liver histology was conducted by HE staining at the indicated times. More than 10 low-power field microscopic examinations were carried out for each slide. We did not observe hepatic necrosis in any of the three groups at 24 h post-d-GalN/LPS injection. We observed moderate hepatic necrosis in the NS and control BMSC groups, whereas only mild hepatic necrosis was observed in the c-Met-BMSC group at 48 h post-d-GalN/LPS injection. At 72 h after d-GalN/LPS injection, moderate or severe hepatic necrosis was observed in the NS and control BMSC groups, whereas only sporadic (spotty) hepatic necrosis was observed in the c-Met-BMSC group (Fig. 6b). Furthermore, the pathological scores were determined using the HAI by blinding of expert pathologists. As shown in Fig. 6c, there were significant differences in HAI scores among the three groups at both 48 and 72 h post-d-GalN/LPS injection (*P* < 0.001). Interestingly, HAI scores in the c-Met-BMSC group were significantly lower than those in the control BMSC group at 48 and 72 h post-d-GalN/LPS injection (*P* < 0.01). These findings strongly suggest that c-Met-BMSCs improved the liver function in ALF induced by d-GalN/LPS and, in some of the cases, even resulted in reversal of the disease allowing rats to recover from ALF.

## Discussion

Transplantation of mesenchymal stem cells (MSCs), liver progenitor cells (LPCs), and hematopoietic stem cells (HSCs) has emerged as a promising therapy to treat various forms of liver disease mainly due to the capacity of these cell types to promote liver regeneration [10, 11]. However, only a small proportion of stem cells appear to be able to migrate to the liver, which largely results in decreased efficacy of their therapeutic use. The main novel findings in this study can be summarized as follows: 1) overexpression of c-Met significantly increased the migration ability of BMSCs in vitro; 2) c-Met-BMSCs homed into ALF-affected rat livers more efficiently than control BMSCs; 3) transplantation of c-Met-BMSCs resulted in significantly increased survival rates and liver function of rats with ALF compared with administration of control BMSCs; and 4) transplantation of c-Met-BMSCs greatly ameliorated liver injury, and the effects of c-Met-BMSCs exceeded those of control BMSCs.

In this study, the ALF rat model was generated by intraperitoneal injection of d-GalN/LPS as previously reported [7]. In recent years, many studies have shown that transplantation of BMSCs could repair injured tissues including those in the liver [12, 13]. Indeed, BMSC transplantation has been considered as a promising approach for the treatment of ALF [9, 14], and has been attributed to the ability of the cells to differentiate into primary hepatocytes and promote liver regeneration [15, 16]. Furthermore, Salomone and colleagues have reported that transplantation of adipose tissue-derived mesenchymal stem cells (ASCs) was effective in treating acetaminophen-induced liver injury. ASCs appear to engraft in the injured liver where they enhance hepatocyte regeneration and inhibit stress and inflammatory signaling [17]. Furthermore, these cells can produce a range of cytokines and growth factors, including insulin-like growth factor-1, vascular endothelial growth factor-1, epidermal growth factor, HGF, and keratinocyte growth factor [18, 19], which have been shown to promote hepatocyte regeneration in situ, suppress inflammatory responses, and inhibit apoptosis of hepatocytes. However, the repair efficacy of BMSC transplantation has been found to be poor [20] mainly due to the low homing efficiency of BMSCs into the injured sites of the liver [4].

HGF is a potent hepatic mitogen produced by liver mesenchymal cells. Zhu et al. [6] have reported that the level of HGF in the liver is significantly upregulated in the rat model of ALF. Our finding that HGF significantly increased after liver injury induced by d-GalN/LPS injection in rats is consistent with previous results. Since HGF has been found to strongly inhibit apoptosis of hepatocytes, elevated levels of HGF may improve the survival rate of mice with ALF. Further studies have shown that HGF also plays an essential role in stimulating liver regeneration against ALF [21, 22]. c-Met is known as the receptor for HGF, and the HGF/c-Met signaling pathway has been demonstrated to participate in different cellular processes such as apoptosis and cell proliferation. Indeed, stimulation of this pathway has shown profound antiapoptosis, antioxidation, and cell proliferation-promoting effects [23, 24]. Moreover, enhancement of HGF/c-Met signaling can promote regeneration of hepatocytes in response to ALF [25, 26]. Recently, a number of studies have shown that the HGF/c-Met signaling pathway can promote cell migration [27, 28]. Therefore, we hypothesized that overexpression of c-Met could promote the homing capabilities of BMSCs to the rat livers affected by ALF, and consequently improve the therapeutic efficacy of BMSC transplantation for the treatment of ALF.

Extensive studies have demonstrated that gene therapy is a promising approach for the treatment of many forms of diseases, and may improve patient care, survival, and outcomes [29, 30]. Viral vectors such as lentivirus, adenovirus, and adeno-associated viral vectors are widely used, mainly because they are highly efficient in introducing target genes into host cells. Compared with adenovirus and adeno-associated viral vectors, lentivirus possesses the ability to transport larger gene fragments thus ensuring earlier protein expression [31]. As a result, lentivirus is an attractive gene delivery system and is widely used in gene therapy [32]. The lentivirus expression vector, originated from the human immunodeficiency virus (HIV), is capable of infecting almost all mammalian cell types. Considering the advantages of the lentivirus as a high-efficiency gene delivery system for gene therapy [33], the lentivirus expression vector was selected in this study to deliver the c-Met gene into BMSCs. Our results validated the stable expression of c-Met protein in BMSCs after transfection.

Notably, the levels of HGF in liver tissue were significantly upregulated at 24 h and 48 h after induced liver injury in the ALF rat model. When the c-Met-BMSC and control BMSC transplantation groups were compared, we found that ALF rats in the c-Met-BMSC transplantation group experienced better therapeutic effects as assessed by survival rates, markers for liver function, and liver pathology. It is plausible that induction of the HGF/c-Met signaling pathway led to augmented c-Met-BMSC migration to the liver and, as a result, a greater number of c-Met-BMSCs were able to home into the rat liver. This theory is supported by our study results, which showed increased migration of BMSCs by in vitro transwell assays. Furthermore, we were able to demonstrate homing capabilities of c-Met-BMSCs to the injured liver compared with control BMSCs using an in vivo imaging system.

Our study does have some limitations, as we could not exclude the possibility that cytokines such as SDF-1 might also be involved in the migration of c-Met-BMSCs [34–36], and an increase in the paracrine effects of c-Met-BMSCs may be responsible for a better effect in comparison with control BMSCS. Further studies are underway in our laboratory to characterize c-Met-BMSC-secreted cytokines in cell culture. In addition, stable expression of a proto-oncogene such as c-Met may lead to increased risk of tumorigenesis. Therefore, it is necessary to regulate exogenous expression of c-Met in the c-Met modified BMSCs by using the tetracycline-on (Tet-On) system [37] prior to its application in future clinical studies.

## Conclusion

In conclusion, our results indicated that overexpression of c-Met promotes homing of BMSCs to the injured liver of rats with ALF, thereby improving the efficacy of BMSC therapy for the repair of ALF.

## Abbreviations

ALF: Acute liver failure
ALT: Alanine transaminase
ASC: Adipose tissue-derived mesenchymal stem cell
AST: Aspartate aminotransferase
bFGF: Basic fibroblast growth factor
BMSC: Bone marrow-derived mesenchymal stem cell
d-GalN: d-Galactosamine
DIR: Deep red fluorescence
DMEM: Dulbecco’s modified Eagle’s medium
ECL: Enhanced chemiluminescence
ELISA: Enzyme-linked immunosorbent assay
FBS: Fetal bovine serum
HAI: Hepatic activity index
HE: Hematoxylin-eosin
HGF: Hepatocyte growth factor
HIV: Human immunodeficiency virus
HRP: Horseradish peroxidase
HSC: Hematopoietic stem cell
LPC: Liver progenitor cell
LPS: Lipopolysaccharide
LT: Liver transplantation
MSC: Mesenchymal stem cell
NS: Normal saline
PET: Polyethylene terephthalate
SD: Standard deviation
SDS-PAGE: Sodium dodecyl sulfate-polyacrylamide gel electrophoresis
TBil: Total bilirubin
Tet-On: Tetracycline-on
WB: Western blot
